# Importance of Retesting for the Final Diagnosis of Post-Stroke Cognitive Impairment

**DOI:** 10.3390/medicina59030637

**Published:** 2023-03-22

**Authors:** Dominik Koren, Miriam Slavkovska, Marianna Vitkova, Zuzana Gdovinova

**Affiliations:** Department of Neurology, Faculty of Medicine, University of P.J. Šafárik and University Hospital of L. Pasteur, Trieda SNP 1, 04011 Košice, Slovakia

**Keywords:** stroke, post-stroke cognitive impairment, prevalence, MoCA test, retesting

## Abstract

*Background and Objectives:* Post-stroke cognitive impairment (PSCI) has been defined as all problems in cognitive function that occur following a stroke. Studies published thus far on the prevalence of PSCI and post-stroke dementia (PSD) have shown conflicting estimates. The aim of this study was screening for cognitive impairment (CogI) in patients with an ischaemic stroke and finding the relationship between CogI (and its changes) and cardiovascular risk factors and imaging procedures—CT/MRI. *Materials and Methods:* We prospectively included patients with an ischaemic stroke admitted in the period from October 2019 to May 2022. In this period, 1328 patients were admitted, 305 of whom met the established inclusion criteria and underwent an examination of cognitive functions using the Montreal Cognitive Assessment (MoCA). Of these, 50 patients appeared for the control examination after 6 months. *Results:* In the retested group, CogI at discharge was diagnosed in 37 patients (74%). In follow-up testing after 6 months, CogI was present in 30 patients (60%). Only arterial hypertension (OR: 15; 95% CI; Pearson r: 0.001), lower education level (less than 13 years) (OR: 9.7; 95% CI 2.0–48.5; Pearson r: 0.002), and higher age were significantly associated with CogI after stroke. *Conclusions:* We established the prevalence of CogI and its course after 6 months in a well-defined group of patients after a mild ischaemic stroke (mean NIHSS: 2 and mean mRS: 1 at the discharge). Our results show that the prevalence of CogI after an ischaemic stroke at discharge is relatively high (74%), and it tends to be a spontaneous reduction. Cognitive functions were changed in 35% of patients. The definition of PSCI was completed in only 24% of individuals. Only an examination several months after a stroke can give us more accurate information about the true prevalence of persistent CogI after a stroke.

## 1. Introduction

Neurocognitive disorders are interesting and extremely important areas of current medicine. In the field of cognitive neuroscience as well as basic and clinical research, the aim is to reveal the physiological principles of cognitive impairment (CogI) and the pathological mechanisms of CogI, to develop preventive procedures, and to provide therapeutic intervention with the potential to slow down and optimally stop the progression of CogI, and it is constantly ongoing. It is estimated that up to 57 million people worldwide currently suffer from neurocognitive impairment, and the annual increase of new cases has reached 10 million and is still increasing, with experts predicting an increase in the global prevalence of neurocognitive impairment to 153 million people in 2050 [1]. From an aetiological point of view, the second most common form (about 15–30% of cases) is represented by vascular cognitive impairment (VCI) [2]. The term VCI is an umbrella term for a wide range of vascular disorders, the consequence of which is CogI. It includes brain ischaemic lesions (stroke), intracerebral haemorrhage, or multifocal cerebral microhaemorrhages due to cerebral amyloid angiopathy (CAA) [3,4]. Based on the level of CogI, VCI can be divided into two categories—mild VCI and major VCI, which is a synonym for vascular dementia (VaD). The authors of the Vascular Impairment of Cognition Classification Consensus Study (VICCCS) proposed the term post-stroke dementia (PSD), defined as immediate and/or delayed cognitive decline that begins after (but within 6 months) of a stroke and that does not recover. PSD can result from different vascular causes, which include large or multiple cortico-subcortical infarcts, small vessel disease with subcortical lacunar lesions, and strategic infarcts [5]. Other authors have also described the term post-stroke cognitive impairment (PSCI) not fulfilling the criteria for major neurocognitive impairment, which means that the functional state is preserved, in contrast to PSD [6]. PSCI has been defined as all problems in cognitive function that occur following a stroke, irrespective of the (stroke) aetiology [7]. 

Physical disability following a stroke is not the only negative consequence limiting the functional state of patients. Despite the well-known frequent incidence of CogI after a stroke, routine cognitive assessment is frequently neglected. Even though the overall annual stroke incidence is decreasing, an increased incidence of strokes in young adults has been registered [8,9]. Therefore, it is important to recognise PSCI by an appropriate assessment protocol in every patient after a stroke, especially in those of a still productive age, because CogI may often be an isolated factor negatively affecting their reintegration to professional life and daily living. PSCI is a major source of morbidity and mortality after a stroke worldwide, but less attention is paid to the fact that it is also a significant factor influencing the return to work, especially in younger patients. In most stroke departments, a good outcome after a stroke is evaluated on the basis of NIHSS and mRS, which, however, take little account of the patients’ cognitive status. Therefore, it may be that stroke survivors with minor functional deficits have difficulty returning to work because of CogI, but their discharge report does not include information about their cognitive status. Insufficient testing of cognitive functions and inconsistent testing methods also account for the variable data on PSCI. 

Studies published thus far on the prevalence of PSCI have shown conflicting estimates, with rates of neurocognitive impairment at 3 months post-stroke ranging from 6% to more than 30%. Inconsistent findings have also been reported on the relationship of PSCI and PSD with risk factors, such as age, low educational attainment, cardiovascular risk factors, or leukoaraiosis [10,11]. Currently, a uniform definition and classification of PSCI, timing of diagnosis, approaches to neurocognitive assessment, and duration of follow up after stroke are not clearly established.

The primary objective of this study was to estimate the prevalence of CogI early after an ischaemic stroke (at discharge) and 6 months after stroke, and to study whether the cognitive status of patients changed over this time. We further analyzed the relationship of CogI with the risk factors and with the pathological neuroimaging findings. 

## 2. Materials and Methods

### 2.1. Study Participants

**Figure 1:**
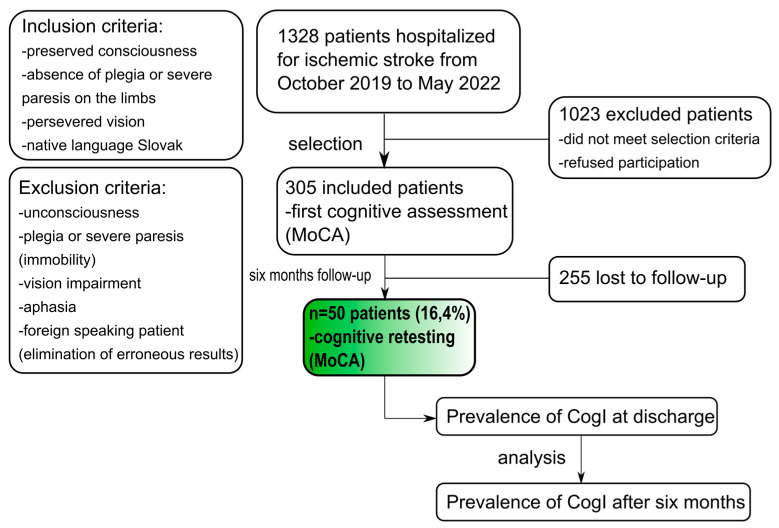
Flowchart of patients’ recruitment with selection (inclusion and exclusion) criteria.

We prospectively included patients with an ischaemic stroke admitted to the Neurological Department of the UPJŠ Faculty of Medicine and L. Pasteur University Hospital in Košice in the period from October 2019 to May 2022. The study was approved by the Ethics comittee of L. Pasteur University Hospital in Košice and complied with the Declaration of Helsinki (World Medical Association). Only patients who fulfilled selection criteria and underwent a control examination of cognitive functions after 6 months were included in the final analyses (Figure 1).

### 2.2. Evaluation Tools

Testing of cognitive functions was performed by using the Montreal Cognitive Assessment (MoCA) [9,12]. Testing was carried out by trained personnel (a neurologist and a psychologist with an official certificate for the implementation of the MoCA test) according to the uniform official protocol and testing rules officially available online at www.mocatest.org. To achieve a uniform, objective, and adequate assessment of the cognitive status of patients, some items of the MoCA test were adapted specifically for the needs of the examination in the conditions of the Slovak Republic, mainly taking into account the mother tongue. The presence of mild CogI was assessed by the official cut-off score of the MoCA test (<26 points). The severity of CogI was classified as mild (25–18 points), moderate (17–10 points), and severe (9 or less points) according to the results of the MoCA test.

Data on demographic characteristics (age, gender, educational attainment), risk factors (arterial hypertension, diabetes mellitus, dyslipidaemia, atrial fibrillation, hypothyroidism, nicotinism), and neuroimaging findings (acute ischaemic changes on CT/MRI) were obtained from the local hospital information system. 

Stroke severity was assessed using the National Institute of Health Stroke Scale (NIHSS) (minor stroke, 1–4; moderate stroke, 5–15; moderate to severe stroke, 16–20; and severe stroke, 21–42) and functional state was assessed using the modified Rankin Scale (mRS) [13,14].

The TOAST (trial of ORG 10172 in acute stroke treatment) classification was used for the aetiological classification of an ischaemic stroke [15]. Acute ischaemia was evaluated from several points of view. We evaluated the side of the affected hemisphere (left and right) and the symptomatic circulation (anterior and posterior). From an anatomical point of view, we determined 11 anatomical regions of the brain: frontal lobe (cortical, subcortical and periventricular zone), parietal lobe (cortical, subcortical and periventricular zone), temporal lobe (cortical, subcortical and periventricular zone), insular lobe, occipital lobe (cortical, subcortical and periventricular zone), basal ganglia (BGG), thalamus, internal capsule (IC), external capsule (EC), and cerebellum and brainstem (mesencephalon, pons Varoli and medulla).

### 2.3. Study Protocol

Patients who met the specified selection criteria underwent a cognitive functions examination, which was performed between 09:00 and 12:00 on the day of the patient’s discharge from the hospital. All these patients were invited for a follow-up examination after 6 months (Figure 1).

The total point score achieved and the performance in individually tested cognitive domains at discharge and after 6 months were recorded. After two MoCA tests scored over time, the change in cognitive status between the first and follow-up examinations was calculated for each patient. We then evaluated whether this change meant improvement (regression) or deterioration (progression).

### 2.4. Statistical Analysis

All analyses were performed using the IBM SPSS 26. (Manufacturer: IBM; IBM SPSS Statistics 26.0, New Orchard Road, Armonk, NY, USA). Firstly, descriptive statistics were used to calculate the prevalence of CogI at discharge and 6 months after the stroke. The presence of CogI was analysed as binary nominal variable (0 = without CogI; 1 = CogI present). Next, we calculated the proportion of patients in whom the MoCA test score between the first and the follow-up examination changed. To compare the means of total score and individual cognitive domain subscores (scale variables) recorded at discharge and after 6 months, the paired samples T-test was used. The odds ratio (OR) and the Pearson coefficient were calculated to show the cross-sectional relationships between the selected variables (age, gender, education, arterial hypertension, diabetes mellitus, dyslipidaemia, atrial fibrillation, hypothyroidism, nicotinism, previous stroke, stroke aethiology, type of treatment, and anatomical localisation of acute ischaemic lesions). For analysis relationships between CogI and anatomical localisation of acute ischaemic lesions, an evaluation system was used based on the presence of a lesion in a determined anatomical region of the brain (0 = not present; 1 = present). The presence of ischaemic lesions in this region was evaluated by a radiologist. A Bonferroni correction was used for statistically significant *p*-values of multiple comparison tests.

## 3. Results

From the 1328 patients admitted to hospital with an ischaemic stroke, cognitive functions were examined in 305 (66% males; mean age 66.6 ± 10.5 years) individuals who met the selection criteria. CogI detected at discharge was present in 247 (81%) patients, and mild CogI was found in 168 (68%) of the patients and moderate CogI in 79 (32%). Mean NIHSS at admission was 5 (SD ± 4.0) and 2 at discharge (SD ± 1.8), and mean mRS was 2 (SD ± 1.5) and 1 (SD ± 1.2) at discharge.

### 3.1. Basic Characteristics of Patients with Follow-Up Observation

The follow-up examination after 6 months was performed in the cohort of 50 patients (35 men, 15 women; mean age, 63.6 ± 10.9 years) who accepted the invitation for subsequent control of cognitive functions. Most patients had a primary or secondary education (82%, up to 13 years). The follow-up cohort consisted of patients with a mild to moderate degree of a stroke (mean NIHSS at discharge 1.5 ± 1.6) with no or slight disability in the majority of patients (mean mRS at discharge 1.0 ± 1.2). The baseline clinical characteristics of the sample (aetiology of an ischaemic stroke, risk factors, and type of stroke treatment) are shown in Table 1.

### 3.2. CogI in the Follow-Up Cohort 

CogI at discharge was diagnosed in 37 of 50 patients (74%). The mean total score on the MoCA test was 23 points (SD 3.8, min: 14; max: 30). Mild CogI was diagnosed in 31 patients (84%) and moderate in 6 patients (16%), and there was no severe CogI in the group. 

**Figure 2:**
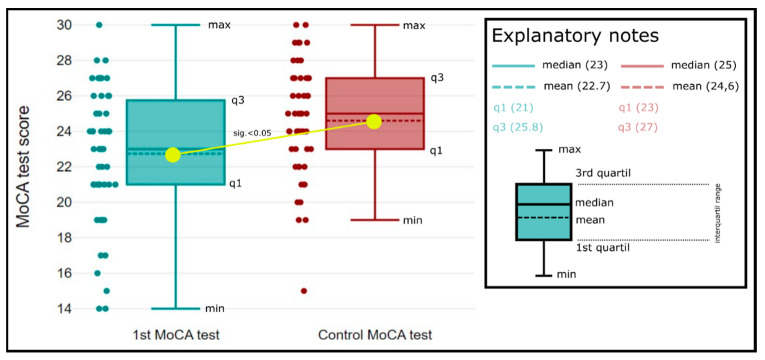
Box-plot diagrams. Comparison of total MoCA score at discharge (green) and 6 months after stroke (red). Mean total MoCA score was significantly increased after 6 months (*p*-value < 0.05).

In follow-up testing after 6 months, CogI was present in 30 patients (60%) with the mean total score in the MoCA test was 25 points (SD 3.2; min:15; max: 30), mild CogI in 29 patients (58%), and moderate in 1 patient (2%). Comparing the means of total MoCA scores detected at discharge and 6 months after the stroke, we found a significant improvement of cognition (Figure 2). 

Analysis of follow-up MoCA scores after 6 months revealed that the MoCA score changed in 35 (70%) patients. The progression of existing CogI (lower score in MoCA) was found in 9 patients, the improvement of CogI (higher score in MoCA) in 22 patients (of whom 11 subjects achieved an improvement to a normal cognitive state), and 4 patients had a newly discovered CogI. The same MoCA score was observed in 15 patients.

**Figure 3:**
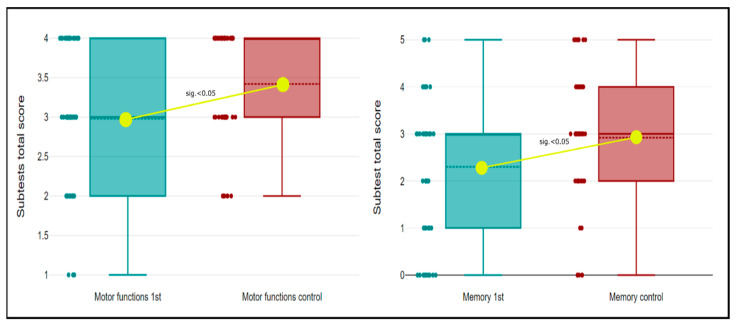
Box-plot diagrams. Comparison of cognitive subtests scores at discharge (green colour) and 6 months after stroke (red colour) in the domain of motor and constructional/visuospatial functions (left) and domain of memory (right). Mean score of subtests was significantly increased 6 months after stroke (*p*-value < 0.05).

The results of the individual domains of the MoCA test—executive functions (TMT test), motor and construction/visuospatial abilities (cube drawing, clock test), language (naming, repetition, phonemic verbal fluency), attention (number repetition, A-test, subtraction), abstract thinking and memory (delayed recall)—are summarised in Table 2. A significant improvement in the MoCA score after 6 months was registered in the domain of motor and constructional/visuospatial functions and in the domain of memory (Figure 3). 

### 3.3. Risk Factors and CogI

Only arterial hypertension (OR: 15) and lower education level (less than 13 years) (OR: 2.5) were significantly related to CogI at discharge. A positive relationship with age was also registered (Pearsons r = 0.317, *p*-value = 0.025) (Table 3). No significant relationship with risk factors and CogI after 6 months was registered.

### 3.4. Imaging Procedures and CogI

Ischaeimc lesions in the territory of the anterior, posterior, and both of the circulations were detected in 35, 13, and 2 patients, respectively. The left hemisphere was affected in 25, the right in 23, and both in two patients. A detailed overview of the anatomical distribution of ischaemic lesions and a summary of CT/MRI findings in relationship to the presence of CogI at discharge are in Table 4. No significant relationship between the presence of CogI after 6 months and the localisation of ischaemias (affected hemisphere, circulation, and brain regions) was registered. 

## 4. Discussion

### 4.1. Prevalence of PSCI

In our study, we found a difference between CogI at discharge and 6 months after a stroke. There is no consensus on the timing of testing cognitive functions, but it is usually within 3–6 months. As far as we know, there have been no works that have been devoted to the comparison of cognitive functions immediately after discharge from the hospital and in an interval of up to 6 months.

Chiti et al. state that repeating the cognitive assessment in the mid-term follow-up of a stroke is crucial for the identification of those patients who would need more and perhaps even specific care interventions. There are patients after the subacute phase after a stroke who are supposedly more stable and may have already experienced a slight spontaneous cognitive recovery [12]. Aam et al., comparing the presence of PSCI 3–18 months after a stroke, found no difference between time points, but executive function and language improved over time [16]. 

Testing at different times after a stroke may account for the different data on stroke prevalence in the literature.

The prevalence of VCI and VD presented in the literature is variable. In Europe (such as the UK, Sweden, France, and the Netherlands), the prevalence of CogI within 3–6 months after a stroke ranges from 24% to 70% according to the MMSE and 96% according to comprehensive neuropsychological test batteries. In other countries, such as Australia, the US, South Korea, and China, the suggested prevalence of VCI is 50%, 58.9%, 69.8%, and 21.8%, respectively [17,18,19,20,21]. Calabrese et al. suggest a two-level assessment procedure consisting of a primary short screening and an in-depth evaluation [22]. 

Optimizing the cognitive testing or psychometric methodology protocol is one of the most important aspects. Based on our testing results, early post-stroke testing overestimates the occurrence of CogI. Therefore, it is advisable to perform the examination of cognitive functions in the period after the stabilization of vital functions, after overcoming the acute period, and after a certain period of physical as well as psychological recovery at discharge (i.e., at the end of hospitalisation). 

In our study, we used the MoCA test as the recommended short clinical psychometric tool for the assessment of patients after a stroke [10,12], and testing was performed at the end of hospitalisation, usually in the morning (from 9:00 a.m. to 12:00 p.m.), when the highest level of concentration and cognitive activity is expected, with retesting after 6 months.

### 4.2. Risk Factors for CogI at Discharge

In our study, we registered only arterial hypertension, low education level, and age as factors significantly associated with CogI at discharge, which is in agreement with other studies [9,23]. The lack of a significant association of other risk factors could be a consequence of the small sample of patients.

Based on the current literature, CogI after a stroke is associated with cardiovascular risk factors, such as arterial hypertension, diabetes mellitus, hyperlipidaemia, atrial fibrillation, valvular defects, and thrombophilic/procoagulant states [11,23,24,25]. In 2020, the Lancet Commission reviewed 12 lifelong risk factors (from early school age/period through adulthood to old age) that influence the development of CogI early after a stroke. The factors include the aforementioned cardiovascular factors and, in addition, other modifiable and preventable factors, such as nicotine addiction, alcoholism, obesity, physical inactivity, depression, hearing disorders, social isolation, level of education, craniocerebral trauma, and air pollution, after they have been determined [26]. 

### 4.3. Factors Modifying Cognition in the Post-Stroke Period

We did not find any statistically significant relationship between changes of cognitive functions (progression, regression) and the monitored risk factors in a period of 6 months after the stroke. Based on our observation of changes of CogI (progression, regression) in the period after the stroke, we suggest that it might be independent of present cardiovascular and demographic risk factors, and the course of cognitive functions in this period depends mainly on cellular and molecular processes following accute brain injury/ischemia. No pharmacological treatment for CogI was initiated in the period after the stroke. We believe this is a very important point for further observation of the natural course of cognitive performance. 

In our study, we observed a statistically significant improvement in the domain of motor and constructional functions and in the domain of memory. The mechanism of CogI after a stroke and its changes over time has not yet been clarified exactly. Executive, motor, and constructional functions are the result of the cooperation of multiple distant brain regions interconnected by numerous functional neuronal networks [27,28]. We assume that this diffuse natural character of neuronal networks involved in executive, motor, and constructional functions allows faster recovery in the period after a stroke, especially in the case of ischaemic lesion of small diameters. In the same way, the function of memory is associated with several functional areas of the brain and their interconnections. The MoCA was designed for testing the function of short-term and working memory, which has an anatomical correlate also in the hippocampus [29,30]. In our group, there was no ischemia located in the hippocampal area in any patient, which we suggest is a possible reason for the significant improvement in the domain of memory. Changes in individual cognitive domains can also be influenced by various exogenous factors (positive and negative) such as social background, social contact, motivation of the patient and cooperation of relatives, depression, anxiety and apathy, and the active life of the patient in the period after the stroke.

Overall, in 22 patients (44%) with CogI at discharge, spontaneous improvement (with no pharmacological impact) of cognitive functions was registered after 6 months. This reflects the fact that the brain has a specific ability to restore cognitive functions. The underlying mechanisms of cognitive function changes after an ischaemic stroke (brain injury) are not known in detail. One of the described phenomena in the early phase after a stroke is a decrease in interhemispheric functional connectivity, and in the period several weeks after stroke, an increased density of functional connections has been described [31,32,33,34]. Reorganisation of the functional connectome of the brain after a stroke within a few months after the insult represents a kind of recovery period associated with dynamic but not fully clarified processes at the cellular and molecular level. This is consistent with recent studies, which show that neurogenesis as a process of neuroplasticity can persist in the adult brain in both physiological and pathological situations, even after a stroke [35,36]. 

### 4.4. CT/MRI Findings and CogI

In most of our patients, ischaemic lesions were of a smaller size (small ischaemias, lacunar ischaemias, or multiple small ischaemic lesions). There was no case with large areas of cortico-subcortical ischaemia. One explanation for the absence of large lesions was the exclusion criteria: patients who could not participate in the follow-up examination due to motor deficits were excluded. Evidence of the smaller size of brain infarcts is reflected in the low mean values of NIHSS and mRS. For future research and statistical analysis, a quantitative measurement based on the volumetry of acute ischaemic lesions is needed for a more accurately correlated statistical analysis. The more practical tool seems to be the Alberta Stroke Programme Early Computed Tomography Score (ASPECTS), which is directly correlated with CogI and by Esmael et al., and it may be considered as a biomarker of PSCI [37].

### 4.5. Clinical Implications

**Figure 4:**
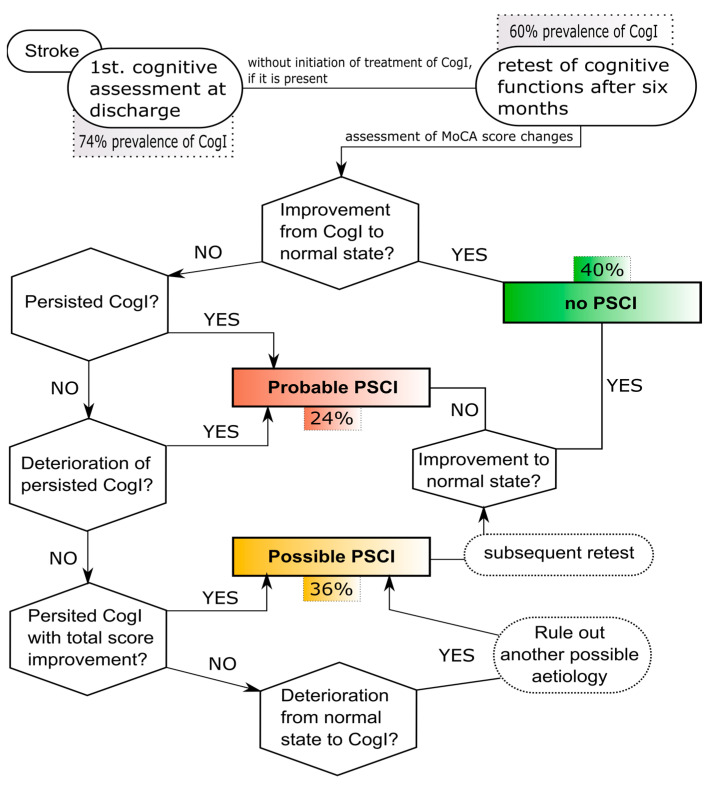
Based on our clinical experience, observation of changes in cognitive functions in patients after Ischaemic stroke, and available prevalence and overview studies, we proposed this algorithm for diagnosis of probable and/or possible PSCI.

Our study showed that the prevalence of CogI in a sample of patients with an ischaemic stroke at discharge was 74% and the prevalence after 6 months was 60%, with a significant change in the total MoCA score suggesting the importance of retesting. Only with time can we say with greater accuracy whether PSCI is present. Based on our results, we can establish the diagnosis of PSCI only after retesting the cognitive functions, and, based on our clinical experience, observation of changes in cognitive functions in patients after ischemic stroke and available prevalence and overview studies were proposed as the algorithm for the diagnosis of PSCI, as shown in Figure 4. According to the proposed algorithm, we registered 12 patients with probable (true) PSCI, 20 patients without PSCI, and 18 patients with possible PSCI who required a follow-up cognitive function examination, which means that they cannot be definitively labelled as PSCI after 6 months. If we counted only patients with probable PSCI, the true prevalence would then be only 24%. A larger prospective study is needed to validate this algorithm.

### 4.6. Study Limitations

The most important limitation of this study is the smaller sample size (number of retested patients). This sample represents 16.4 % from the initially examined 305 patients. All patients signed an informed consent and every patient and their relatives were properly informed and instructed about the importance of control cognitive testing after 6 months, with official recommendation given as a part of the hospital discharge summary. Low patient return rate is multifactorial. Based on the mean NIHSS and mRS scores at discharge, we do not assume that physical disability should be the limiting factor. One of the main reasons that could have contributed to the low retested sample was the pandemic situation related to the SARS-CoV-2 infection, which started shortly after the start of the study. Patients stayed at home due to lockdown measures and general advice not to leave the house, outpatient visits of patients decreased significantly in all countries, including Slovakia, and patients and their relatives were afraid to come to the hospital in general and also just for the cognitive function testing. Another factor is the motivation of the patient and his relatives, the personality of the patient, and also the occurrence of comorbidities, especially depression. A control examination of cognitive functions 6 months after the stroke was performed using the same version of the MoCA test, which could be considered as a possible limitation. However, we assume that the distortion of the results due to the same versions was reduced by a relatively long time gap between the two examinations.

## 5. Conclusions

We found a 74% prevalence of CogI at discharge in patients after a mild ischemic stroke (mean NIHSS: 2 and mean mRS: 1 at discharge), but for a definitive diagnosis of PSCI, retesting of patients after 6 months is necessary, as we saw a change of cognitive function in 35% of the patients. The most frequently affected cognitive domains were memory, language, and motor and constructional (visuospatial) functions. The most significant improvements at 6 months after an ischemic stroke were in the areas of motor and structural function and memory. Following the definition of PSCI, the final diagnosis of PSCI was confirmed in 24% of patients, which represents a substantial reduction in comparison with baseline CogI early after the stroke. The diagnosis of PSCI in addition to the assessment of functional abilities is important, too, for the assessment of a patient’s ability to return to work after a stroke. 

## Figures and Tables

**Table 1:** Baseline characteristics of the analysed group (n = 50).

Demographics	
Age	63.6 years (SD ± 10.9 years)
Gender	35 men
Low education level (<13 years)	41 (82%)
**Risk Factors**	**Prevalence: Number of Patients (%)**
Arterial hypertension	42 (84)
Dyslipidaemia	30 (60)
Nicotinism	23 (46)
Atrial fibrillation	7 (14)
Diabetes mellitus	6 (12)
Hypothyroidism	5 (10)
Stroke	5 (10)
**Stroke Severity**	**Mean (SD)**
NIHSS at discharge	2 (SD ± 1.8)
mRS at discharge	1 (SD ± 1.2)
**Affected Circulation**	**Number of Patients (%)**
Anterior circulation	35 (70)
Posterior circulation	13 (26)
Both	2 (4)
**Stroke Aethiology**	**Number of Patients (%)**
LVD	14 (28)
SVD	11 (22)
CE	8 (16)
Undetermined	16 (32)
Other	1 (2)
**Therapy**	**Number of Patients (%)**
IVT	3 (6)
TE	4(8)
IVT + TE	5 (10)
APT	39 (78)

AF = atrial fibrillation; AH = arterial hypertension; APT = antiplatelet therapy; CE = cardioembolic; DLP = dyslipidaemia; DM = diabetes mellitus; IVT = intravenous thrombolysis; LVD = large vessel disease; mRS = modified Rankin Scale; NIHSS = National Institutes of Health Stroke Scale SVD = small vessel disease; TE = thrombectomy; stroke = history of previous stroke.

**Table 2:** Comparison of mean scores and prevalence of dysfunction in individual cognitive domains at discharge and 6 months after stroke.

Cognitive Domain	Prevalence of CogI at Discharge [n (%); Mean MoCA Score (SD)]	Prevalence of CogI after Six Months [n (%); Mean MoCA Score (SD)]	*t*-Test *p*-Value
Executive functions	21 (43%); 0.43 (0.5)	19 (38%); 0.37 (0.5)	0.371
Motor and constructional functions	34 (68%); 3.0 (0.9)	24 (48%); 3.4 (0.7)	0.000 *
Language & speech	35 (70%); 4.6 (1.3)	37 (74%); 4.7 (1.1)	0.542
Attention	24 (48%); 5.04 (1.2)	23 (46%); 5.31 (0.9)	0.065
Abstract thinking	19 (38%); 1.56 (0.6)	16 (32%); 1.6 (0.6)	0.687
Memory	39 (78%); 2.3 (1.54)	33 (66%); 2.9 (1.43)	0.004 *

n = number of patients; % = percent from all analysed sample; SD = standard deviation, *t*-test = two-tiled Students test, *p*-value < 0.05, * = statistically significant change.

**Table 3:** Summary of correlation analysis between presence of CogI at discharge and selected variables (risk factors, type of the treatment, and stroke aethiology).

Presence of CogI at Discharge vs. Risk Factors			
Factors	OR, 95% CI	Pearsons r	*p*-Value
Education (<13 years)	9.7 (2.0–48.5)	0.434	0.002 *
Age		0.317	0.025 *
AH	15 (2.5–90.2)	0.488	0.001 *
AF	0.9 (0.2–5.1)	−0.024	0.867
DLP	1.4 (0.4–5.1)	0.074	0.599
DM	1.1 (1.0–1.4)	0.219	0.122
stroke	1.2 (1.0–1.3)	0.198	0.162
smoking	1 (0.23–3.53)	−0.002	0.990
hypothyroidism	0.5 (0.1–3.3)	−0.106	0.452
**Presence of CogI at Discharge vs. Aetiology of Stroke**			
**Aetiology**	**OR, 95% CI**	**Pearsons r**	***p*-Value**
LAA	1.4 (0.3–6.1)	0.065	0.646
lacunar	0.9 (0.2–4.2)	−0.015	0.913
CE	0.5 (0.1–2.6)	−0.114	0.418
UE	1.1 (0.2–4.8)	0.013	0.928
**Presence of CogI at Discharge vs. Treatment**			
**Treatment**	**OR, 95% CI**	**Pearsons r**	***p*-Value**
IVT/TE	0.6 (0.2–2.6)	−0.020	0.893
**Progression of CogI (Decline in MoCA Score after 6 Months) vs. Risk Factors**			
**Risk Factors**	**OR, 95% CI**	**Pearsons r**	***p*-Value**
education	2.9 (0.6–15.0)	0.187	0.186
AH	0.6 (0.1–3.6)	−0.08	0.574
AF	0.7 (0.1–6.9)	−0.039	0.783
DLP	0.5 (0.1–2.0)	−0.149	0.293
DM	0.8 (0.7–0.9)	−0.173	0.221
smoking	3.0 (0.6–12.9)	0.194	0.170
stroke	0.8 (0.7–0.9)	−0.156	0.269

AF = atrial fibrillation, AH = arterial hypertension, DLP = dyslipidemia, DM = diabetes mellitus, IVT = intravenous thrombolysis, TE = thrombectomy, LAA = large artery atherothrombosis, UE = undetermined aetiology, CE = cardioembolic aetiology. * = statistically significant change.

**Table 4:** Characteristics of analyzed group (n = 50) according to localization and number of new ischaemic lesions on CT/MRI examination.

CT/MRI Findings of Acute Ischaemic Lesions			
Anatomical Region	Number of Patients		%
Frontal lobe	12		24
Temporal lobe	14		28
Parietal lobe	13		26
Occipital lobe	9		18
Insula	8		16
Basal ganglia	11		22
Internal capsule	5		10
External capsule	6		12
Thalamus	4		8
Brainstem	6		12
Cerebellum	7		14
**Number of Affected Regions**	**Number of Patients**		**%**
1	18		36
2	15		30
3	5		10
4	4		8
5	2		4
6	1		2
**Relationship between Presence of CogI at Discharge and Localisation of Ischaemic Lesion**			
**Symptomatic Circulation & Hemisphere**	**OR**	**Pearson r**	***p*-Value**
Anterior	1	0.01	0.944
Posterior	1	0.040	0.775
Right	2	−0.319	0.154
Left	4	0.276	0.072
Both	0.6	0.624	1289
**Affected Regions**	**OR**	**Pearson r**	***p*-Value**
Frontal lobe	1.2	0.094	0.506
Parietal lobe	1.3	0.168	0.234
Temporal lobe	1.2	0.138	0.329
Occipital lobe	1.1	0.078	0.580
Insula	1.6	0.239	0.091
Basal ganglia	1.5	0.236	0.096
Internal capsule	1	0.046	0.747
External capsule	2.4	0.342	0.165
Thalamus	1	0.007	0.962
Cerebellum	1	0.024	0.867
Brainstem	0.91	0.079	0.578

## Data Availability

The datasets generated and analyzed during this study are not publicly available due to a lack of participants’ agreement.
